# Supplementary figures and images for: Pharmacodynamic Effects of Standard versus High Caffeine Doses in the Developing Brain of Neonatal Rats Exposed to Intermittent Hypoxia
Source: Int J Mol Sci. 2021 Mar 27;22(7):3473. doi: 10.3390/ijms22073473 (PMC8037517; doi:10.3390/ijms22073473)

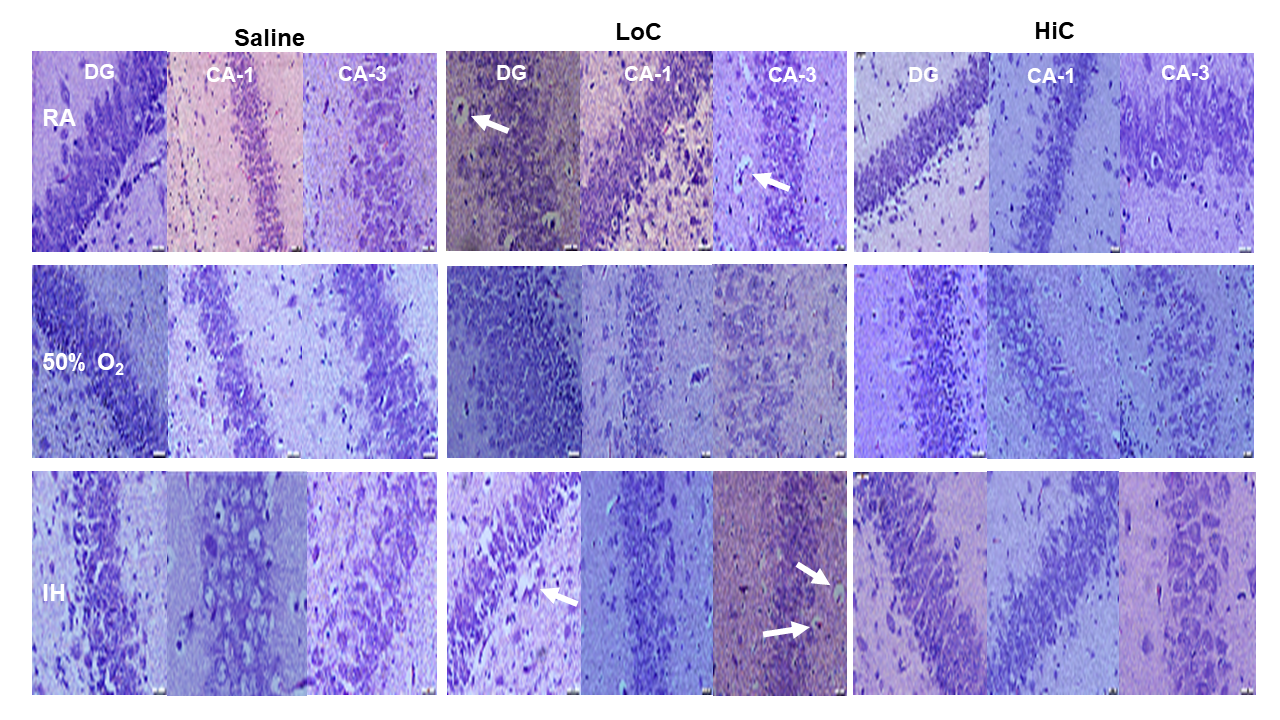

Supplement: Supplementary file 1 [file ijms-22-03473-s001.zip › Soontarapornchai P_IJMS_Supplemental Figures/Slide1.TIF]

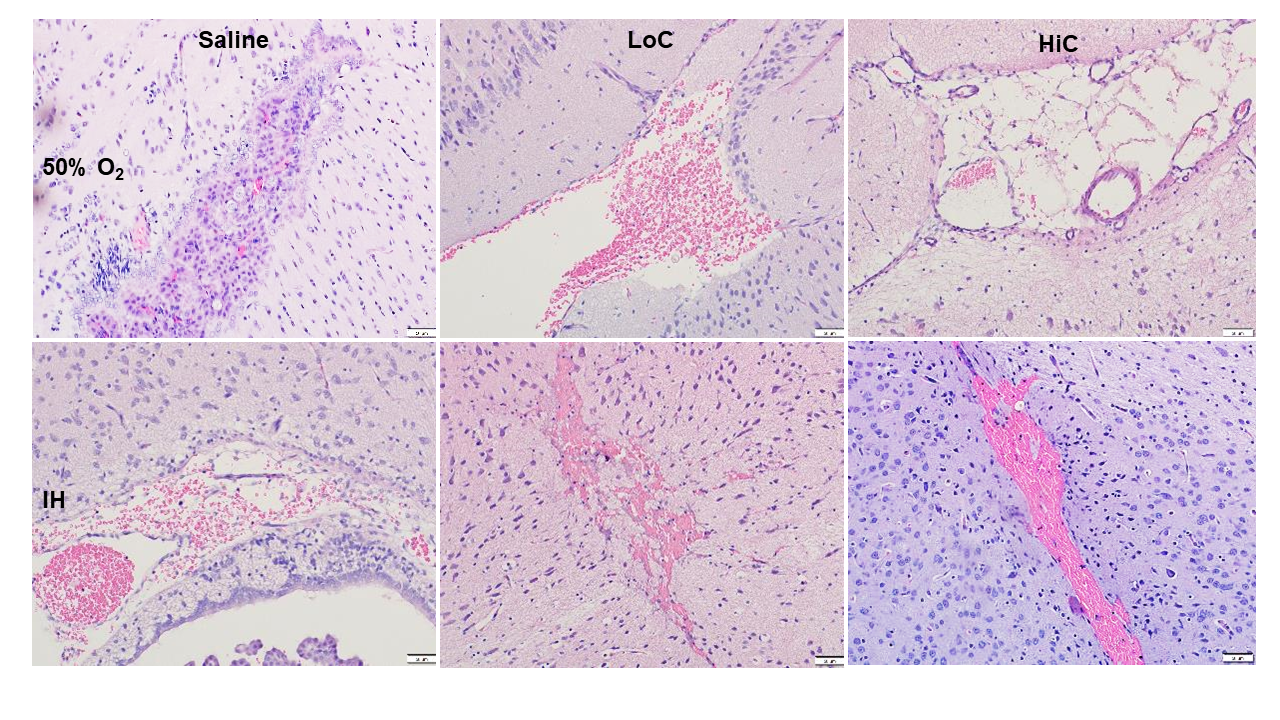

Supplement: Supplementary file 1 [file ijms-22-03473-s001.zip › Soontarapornchai P_IJMS_Supplemental Figures/Slide2.TIF]
